# Electrification of Selective Catalytic Liquid Organic Hydrogen Carriers: Hydrogenation and Dehydrogenation Reactions

**DOI:** 10.1021/acsomega.3c06738

**Published:** 2024-01-29

**Authors:** Anja Sedminek, Blaž Likozar, Sašo Gyergyek

**Affiliations:** † Department for Material Synthesis, Jožef Stefan Institute, Jamova 39, 1000 Ljubljana, Slovenia; ‡ Faculty of Chemistry and Chemical Engineering, University of Maribor, Smetanova 17, 2000 Maribor, Slovenia; § Department for Catalysis in Chemical Reaction Engineering, National Institute of Chemistry, Hajdrihova 19, 1000 Ljubljana, Slovenia

## Abstract

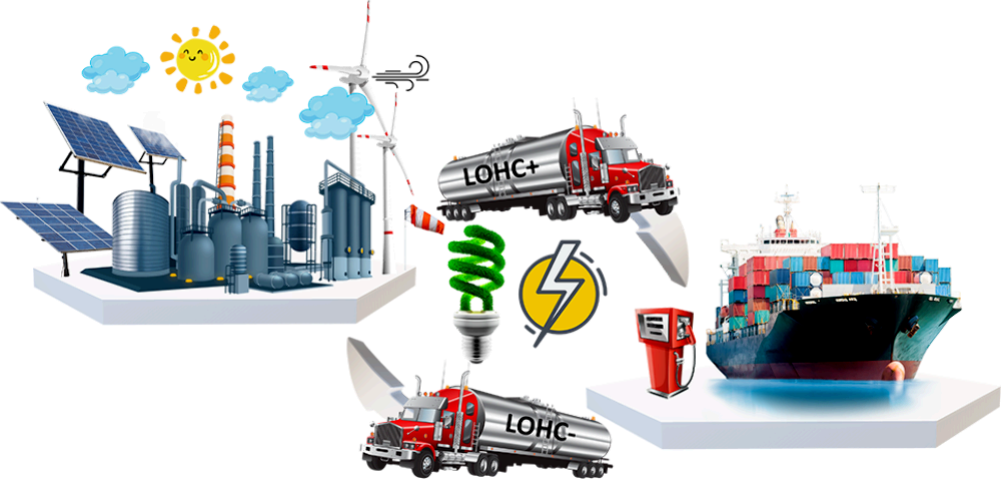

The development of efficient, chemical hydrogen-storage materials is one of the greatest technical challenges for the coming hydrogen-based economy. Analyzed liquid organic hydrogen carriers (LOHCs), which bond, store, and release the H_2_ molecules through catalytic hydrogenation, cracking, and dehydrogenation cycles, are being considered as an alternative, functional option. The search for a highly industrialized reactive production process, coupled with the use of renewable electrical energy, has encouraged the consideration of characteristic stand-alone methods (such as microwave-assisted surface reactions, an increase in the rates by magnetic heating systems, electrocatalysis, variable photochemical manufacturing, and plasma). This mini review aims to highlight, assess, and critically evaluate these recent advances in the electrification of LOHC-related plant technologies. Besides base storing vectors, such as methanol, formaldehyde, and formic acid derivatives, reversible cycling compounds, i.e., benzene, toluene, polycyclic dibenzyl toluene (DBT), carbazole, and indole, are given an overview. These all compete with, for example, ammonia. Specific design methodologies, such as density functional theory (DFT), kinetics, mass-transfer phenomena, etc., are discussed, whether these were studied or the subject of modeling. Lastly, quantitative structure–performance relationships are correlated for activity, selectivity, and stability, where the latter was possible.

## Introduction

Supply disruptions and climate change are forcing us to find safe and practical ways to store and transport renewable energy. As alternatives to fossil fuels, solar and wind energy represent attractive renewable sources. However, due to their intermittent production of electricity, energy-storage systems are essential for their viability. Such systems fall into various categories (chemical, electrochemical, electrical, mechanical, and thermal), and each has its benefits and limitations.^1,2^ In addition, in a recent article, Centi emphasizes that the electrification of chemical production is a major challenge for innovation in the chemical industry and a requirement to achieve the goal of net-zero emissions in the future.^3^

**Figure 1:**
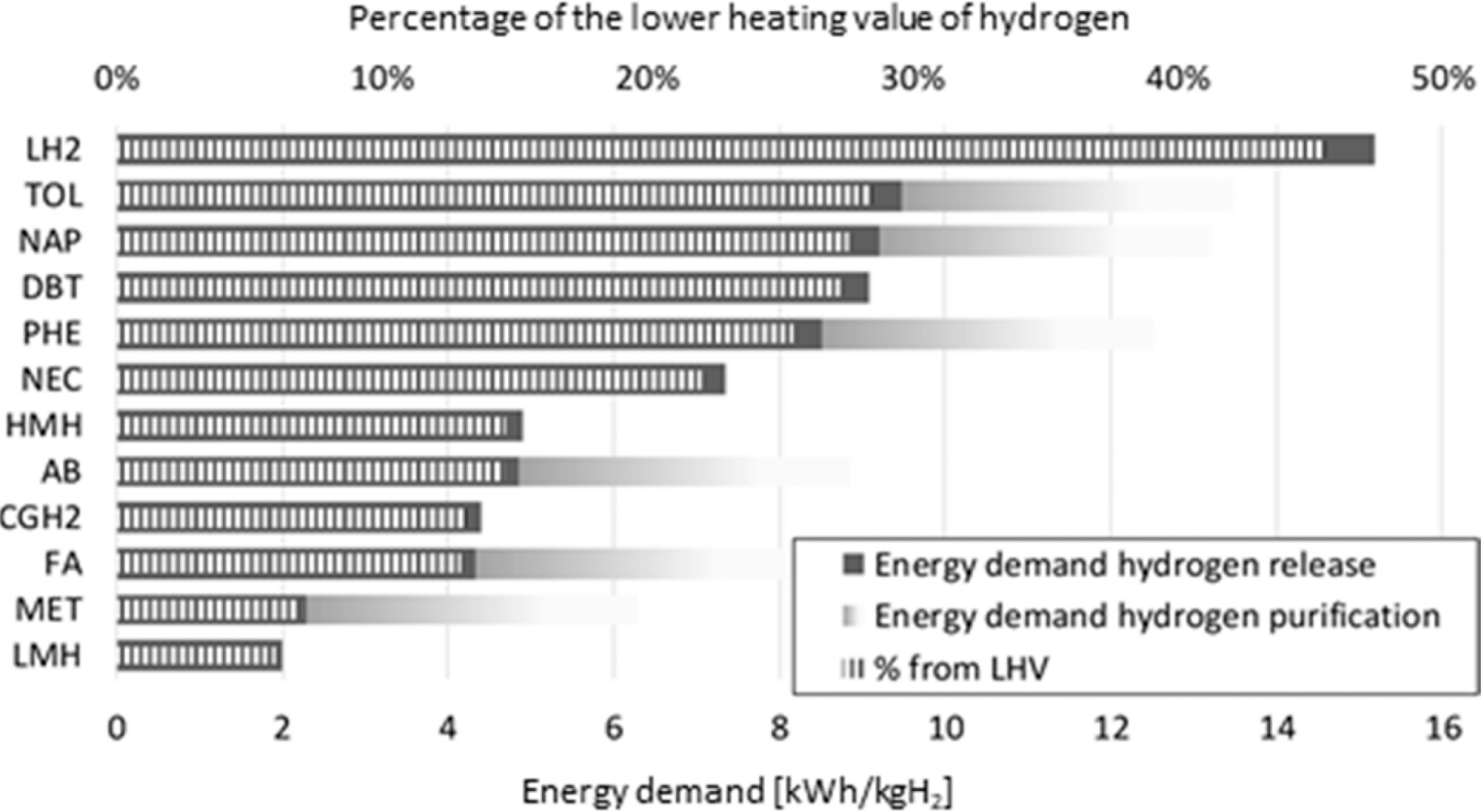
Energy demand for hydrogen storage and its percentage of the lower heating value (LHV) of hydrogen (LH_2_, liquefied hydrogen; TOL, toluene; NAP, naphthalene; DBT, dibenzyl toluene; PHE, phenazine; NEC, *N*-ethylcarbazole; HMH, high-temperature metal hydride; AB, 1,2-dihydro-1,2-azaborine; CGH_2_, compressed hydrogen gas; FA, formic acid; MET, methanol; LMH, low-temperature metal hydride). Reprinted with permission from ref (4). Copyright 2019 Elsevier.

The steadily expanding utilization of carbon-based fuels will likely be partially replaced by hydrogen due to its advantageous properties and the absence of GHG emissions during its use. Nevertheless, conventional hydrogen storage has many challenges associated with compression and liquefaction processes. In particular, there are safety concerns related to the high pressure (700 bar) and low temperature (−252 °C). Its low storage density under ambient conditions complicates its usage, transportation, boil-off issues (−241 °C), and high economic and energetic efforts to store it. According to Figure 1, the energy demand for liquefied H_2_ is by far the highest, due to the energy-intensive liquefaction process.^4−6^ A promising system for hydrogen storage is the use of hydrogen hydrates. However, to achieve storage without a promoter, strict operating conditions must be met, such as a pressure of more than 100 MPa and a temperature of less than 190 K. The main challenges in this context are optimizing the storage capacity, improving the hydrogen charge rate in this storage medium, and ensuring sustained and reliable cyclic performance over extended periods of time.^7^

**Figure 2:**
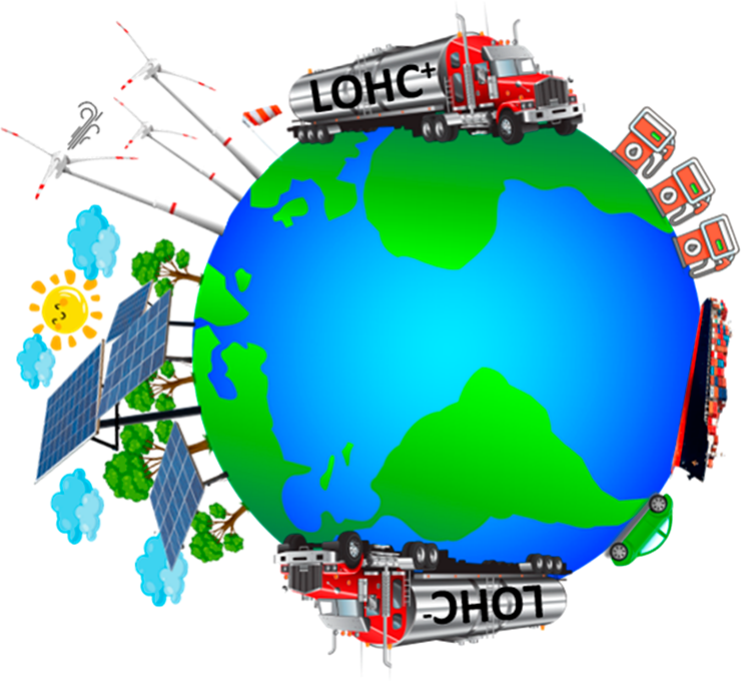
Demonstration of LOHC as a fuel in the future. LOHC+ and LOHC– stand for the hydrogen-rich and hydrogen-lean forms of LOHC, respectively.

An alternative solution for hydrogen storage and transport is a liquid organic hydrogen carrier (LOHC) system, which has enjoyed increasing interest in recent decades. By definition, LOHCs are organic compounds that are in the liquid state under ambient conditions.^1^ There are many critical drivers for this development, mainly; (i) decentralization because of the improved availability of renewable energy sources in locations with low population and thus low energy demand; (ii) coupling with electrolyzer technologies; (iii) the huge interest in green, carbon-neutral, emission-free technologies; and (iv) the compatibility with the existing gasoline infrastructure (Figure 2) for its use as a fuel (lorries, ships, farms). Consequently, hydrogen can be transported via LOHC based on two steps: exothermic hydrogenation (storage of hydrogen) and endothermic dehydrogenation (release of hydrogen) of the LOHC molecule. It is worth noting that both steps can be carried out at the same temperature by regulating the partial pressure of hydrogen. LOHC charging occurs under high hydrogen pressures (above 20 bar), while hydrogen release occurs under low hydrogen pressures (below 5 bar).^2^ The performance of the LOHC system is strongly dependent on the catalyst, the reactor configuration, and the process of (de)hydrogenation (thermal, MW-assisted, electrified, etc.).^1,2^

There are some critical issues concerning LOHC candidates and some key aspects of the LOHC system that should be considered when studying the potential of LOHC, which should possess the following properties:

- low melting point (< –30 °C) of all compounds
- high boiling point (>300 °C) for simplified H_2_ purification
- high hydrogen-storage capacity (>56 kg/m^3^, >6 wt %)
- low heat of H_2_ desorption (42–54 kJ/mol H_2_) to enable low dehydrogenation temperature at 100 kPa H_2_ pressure (<200 °C)^8^
- high selectivity toward (de)hydrogenation for long life cycles and avoiding alternative decomposition pathways, which enables repeated reversibility of the system
- compatibility with the existing infrastructure for fuels
- low production costs and good technical availability
- nontoxic during transportation and use^1,2,5^

**Figure 3:**
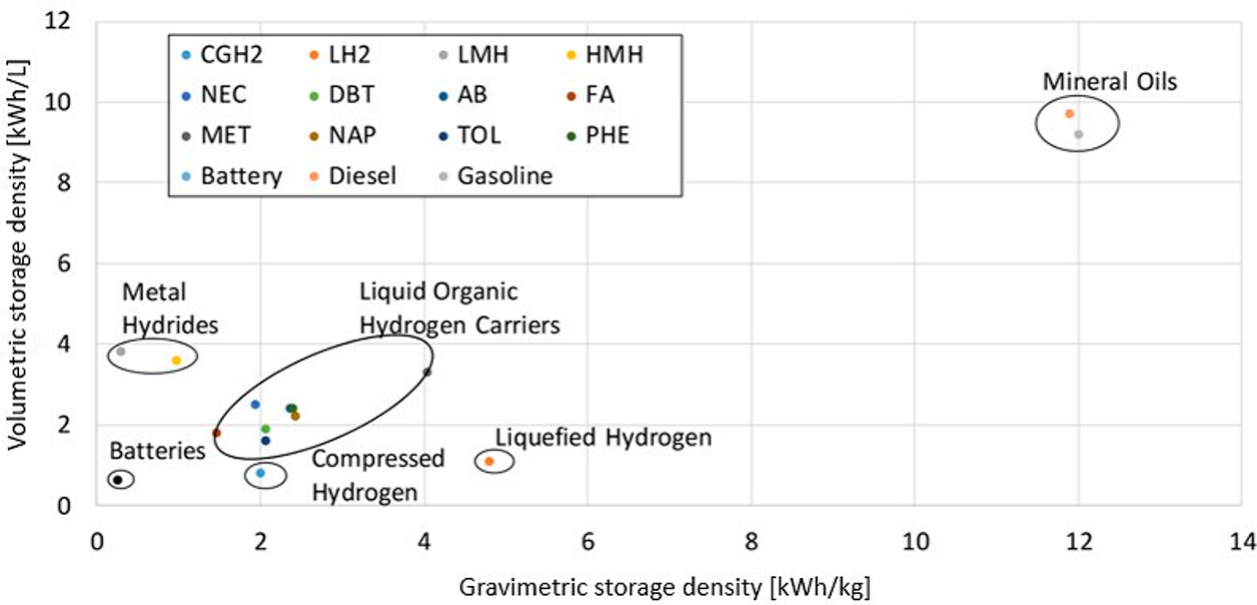
Comparison of volumetric and gravimetric storage density of different hydrogen-storage options (CGH_2_, compressed hydrogen gas; LH_2_, liquefied hydrogen; LMH, low-temperature metal hydride; HMH, high-temperature metal hydride; NEC, *N*-ethylcarbazole; DBT, dibenzyltoluene; AB, 1,2-dihydro-1,2-azaborine; MET, methanol; NAP, naphthalene; TOL, toluene; PHE, phenazine). Reprinted with permission from ref (4). Copyright 2019 Elsevier.

Figure 3 shows the volumetric and gravimetric storage density, i.e., the energy that can be stored per liter and per kilogram, respectively, for different hydrogen-storage options. LOHCs offer a compromise between good gravimetric and sufficient volumetric energy density, compared to the other storage options. The volumetric energy density is comparable to that of methanol at 4 kWh L^–1^. However, the values for the mineral oils (diesel, gasoline) remain higher.^4,5^

Based on the chemical structure and utilization purposes, a LOHC can be categorized into different sections, mainly in cycloalkanes, N-heterocycles, and groups of other, uncategorized, promising LOHCs (Table 1). At the beginning, researchers mostly focused on cycloalkanes. These have favorable properties for hydrogen supply on a large scale and over long distances. This is mainly due to their cost-effectiveness, the high purity of the hydrogen produced, and their abundance.^9^ Despite all the advantages, however, there are major drawbacks for practical applications; i.e., the dehydrogenation of cycloalkanes to their corresponding aromatics occurs at relatively high temperatures (around 300–350 °C) due to the unfavorable enthalpy (approximately 65 kJ/mol H_2_) changes.^10,11^ The issue to be solved was the deactivation of the catalyst due to coking, which could be effectively overcome by the addition of alkali or alkaline-earth metals.^12^ The use of suitable catalysts can mitigate the kinetic lowering of the operating temperature for alkane–arene pairs. However, a thermodynamic improvement can only be achieved by changing the composition.^13^ As Pez et al.^14^ proposed, incorporating heteroatoms (N, O, P, B) into LOHCs lowers dehydrogenation enthalpy to the value of approximately 52 kJ/mol H_2_. Although the thermodynamic properties have been optimized, there are still issues that need further investigation, for example, toxicity, cost, stability, etc. Some of the N-heterocycles are not in the liquid phase after the (de)hydrogenation reaction under ambient conditions, which might also not be favorable for certain aspects.^15,16^ For many practical reasons, commercial solutions and current developments are all still based on pure hydrocarbon-based LOHCs. There are also a number of other promising hydrogen carriers (methanol, formic acid, ethylamine, furan derivatives, etc.) that should be considered. However, the dehydration enthalpies of the aliphatics are high. In addition, some short alkanes exist in the gaseous state under ambient conditions.^15^

**Table 1:**
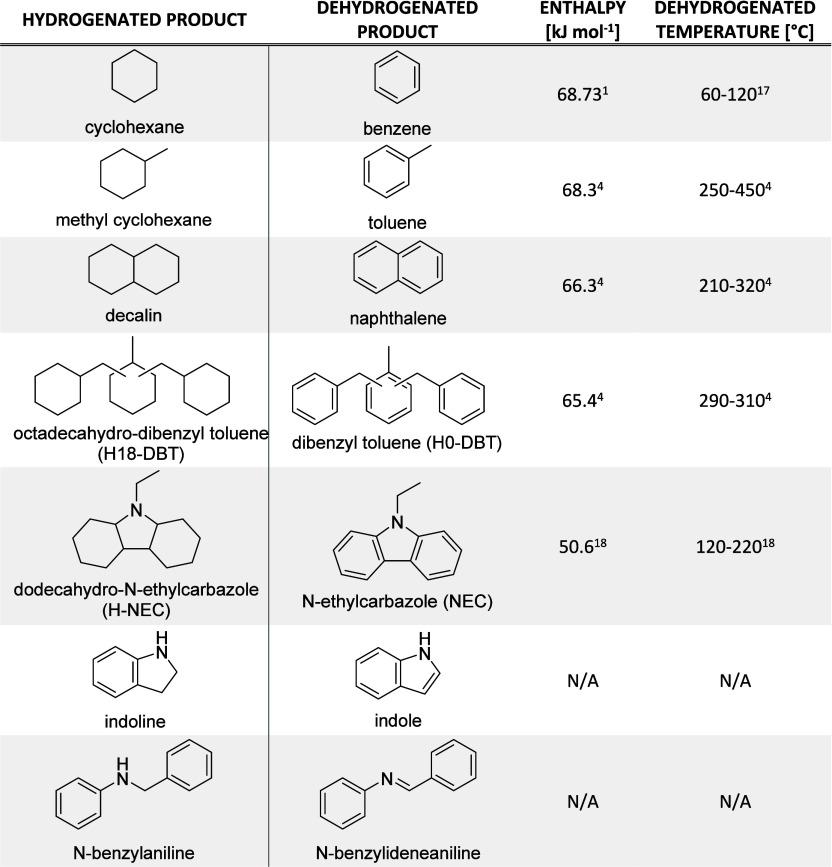
Typical Examples of Potential LOHCs (Hydrogenated and Dehydrogenated Products)^17,18^

There are numerous publications that have examined the conventional (thermal) catalysis of various LOHC systems and techno-economic analyses, etc. Surprisingly, there are no review papers dealing with electrified catalysis, which we believe to be a promising field. Despite the lack of such articles, reviews are important to facilitate traceability in this field. We will present and critically evaluate the electrified storage of hydrogen in different LOHC systems. We target electrified processes because the search for a highly efficient production process combined with the desirable use of renewable energy has led to the development of alternative methods powered by electricity, such as microwave (MW)-, plasma-, and magnetic-heating-assisted catalysis, as well as electrified processes based on electron exchange such as electrochemical catalysis and electrolysis.

## Applications

In the design of a LOHC system, a suitable catalyst for the (de)hydrogenation reaction is essential. This catalyst typically consists of NPs of noble metals such as palladium (Pd),^8^ platinum (Pt), ruthenium (Ru),^20^ and rhodium (Rh)^21^ dispersed on a support material with a high surface area, such as carbon, titania, silica, alumina, or ceria. Alternatively, materials with high porosity, such as zeolites or metal–organic frameworks (MOFs), can be used as the support. The catalyst needs to be highly efficient, active, stable, and reusable to make the LOHC system work effectively.^1^

### Electrified Processes Based on Electron Exchange

**Figure 4:**
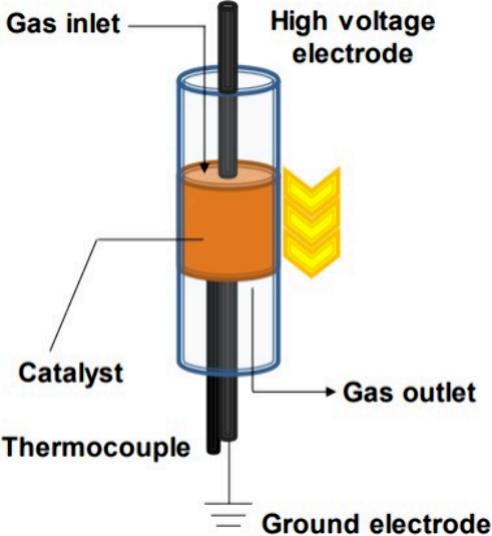
Catalyst bed setup for electrocatalysis reprinted from ref (22). This figure is licensed under a Creative Commons Attribution-NonCommercial 3.0 Unported License.

Electrocatalysis can be defined as heterogeneous catalysis, where the electrochemical/redox reaction occurs at the interface of the electrode and the electrolyte. The electrode represents both roles as an electron donor/acceptor and a catalyst. The catalysis is usually performed in a simple electrochemical cell using a three-electrode system consisting of the working, counter, and reference electrodes. Some researchers perform electrocatalysis directly in a fixed-bed flow-type reactor with two stainless-steel electrodes inserted on the top and bottom sides of the catalyst bed (Figure 4). For the reaction, a current is applied between the electrodes.

Electrocatalytic technologies are very attractive because, by adjusting the electric field, the direction and the rate of the reaction can be controlled at ambient pressure and temperature by changing the activation energy. In electrocatalysis, there is still much room for improvement to develop highly efficient, low-cost, and long-term-stable electrocatalysts. For the characterization of the catalysts and the reaction mechanisms the following methods are the most used: cyclic voltammetry (CV) and linear sweep voltammetry (LSV).^23^

Recently, Driscoll et al.^24^ focused on the potential application of redox catalysis for the dehydrogenation reaction of two model substrates, N-benzyl aniline and indoline, used as a potential LOHC. They presented the preliminary results of catalytic current measurements as a function of different parameters (redox catalyst, base concentration, and base strength). They compared four different catalysts (2,3-dichloro-5,6-dicianobenzquinone - DDQ, ferrocene, decamethylferrocene, and diamino ferrocene) with both substrates. In general, redox catalysis can diminish the overpotential of the dehydrogenation reaction. However, it was observed that adding a weak or strong base to the reaction mixture is necessary. Subsequently, the catalysis occurs through outer-sphere electron transfer via two possible reaction mechanisms, an ECE (electrochemical–chemical–electrochemical) or CEE (chemical–electrochemical–electrochemical), depending on the combination of the catalyst/substrate/base. Quinone appears to catalyze the N-benzyl aniline via a hydride-transfer mechanism under stoichiometric conditions. On the other hand, a ferrocene catalyst exhibits poor activity for the indoline via outer-sphere electron transfer through the ECE mechanism. Furthermore, there was no conversion of N-benzyl aniline without a significant excess of imidazole as a base. Very similar results were obtained in the dehydrogenation of indoline where ferrocene’s derivates were used as the catalyst. The strong base (1,1,3,3-tetramethyl guanidine) was essential for successfully completing the reaction via the CEE mechanism. The author published very similar results in another article.^25^ To summarize, they emphasize that the experiments performed still require considerable optimization to produce efficient dehydrogenation reactions at practical voltages for fuel-cell operation.

**Figure 5:**
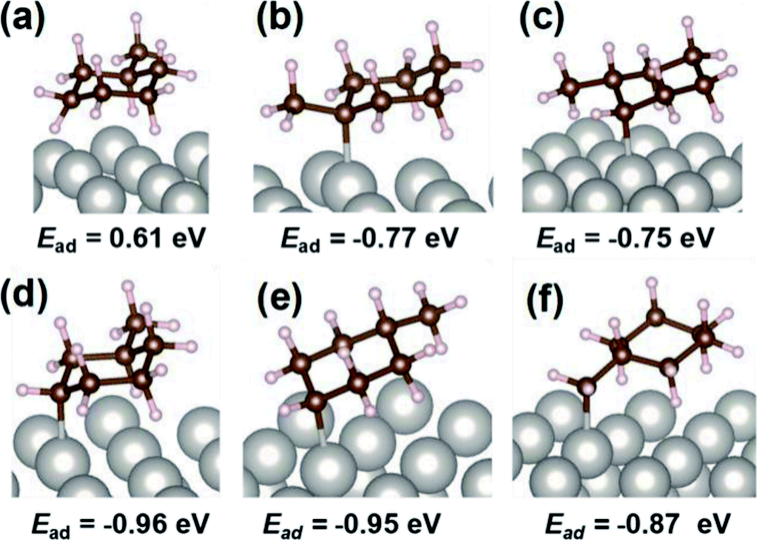
Calculated optimized structure and adsorption energy with DFT calculation for MCH physisorption and C_7_H_13_ chemisorption at various positions: (a) MCH physisorption, (b) α-position, (c) β-position, (d) γ-position, (e) δ-position, and (f) methyl-group. This figure is reprinted from ref (22) and licensed under a Creative Commons Attribution-NonCommercial 3.0 Unported License.

Takise et al.^22^ studied the dehydrogenation reaction of methylcyclohexane (MCH) to toluene using 3 wt % Pt/CeO_2_. The catalytic activity of the latter, usually carried out at temperatures higher than 623 K, was high, even at 423 K. The catalysis was promoted by an electric field placed across a fixed-bed flow reactor. Some interesting peaks belonging to chemisorbed C_7_H_13_ species in the β and δ positions were observed by diffuse reflectance infrared Fourier-transform spectroscopy (DRIFTS) (Figure 5). DFT calculations noted that these positions are favorable for the collision of the protons with hydrogen from MCH. Additionally, toluene hydrogenation was inhibited, since toluene desorption was favored in the electric field.

**Figure 6:**
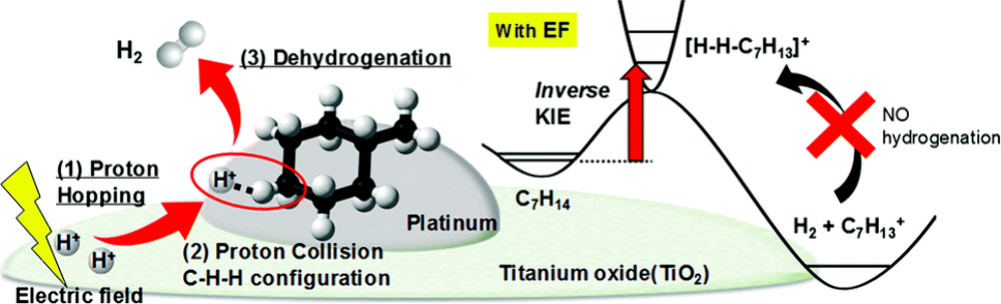
Mechanism of MCH dehydrogenation over Pt/TiO_2_ in an electric field (EF). KIE stands for the kinetic isotope effect. This figure is reprinted from ref (26) and licensed under a Creative Commons Attribution-NonCommercial 3.0 Unported License.

Takise et al.^26^ continued their work on MCH with Pt catalysts. Using DRIFTS analyses, it was found that Pt/CeO_2_ was not suitable as the adsorption amount of toluene was significant compared to Pt/TiO_2_, where only weak peaks characteristic of toluene were detected. A suitable catalyst, Pt/TiO_2_, was used for the dehydrogenation of MCH with and without the application of an electric field. In general, an increase in the temperature leads to a higher hydrogen yield regardless of the presence of the electric field. The effect of the latter was pronounced at lower temperatures, with a hydrogen yield of 17.9% obtained at 423 K in a kinetic regime. It seems that the role of the proton species, studied by the inverse kinetic isotope effect (Figure 6), is essential due to the formation of the C–H(D)–H(D). The latter is formed by the collision of the proton with the H (or D) atoms of MCH. Consequently, the dehydrogenation has a higher activation energy (*E*_a_), making it challenging to proceed. Furthermore, X-ray photoelectron spectroscopy (XPS) measurements demonstrated that more Pt was reduced to metallic Pt(0) after applying the electric field, which leads to weaker interactions between Pt and the π-coordination of toluene. They concluded that the Pt/TiO_2_ catalyst selectively promotes MCH dehydrogenation by proton hopping in the electric field at low temperature. In their research, Kosaka et al.^27^ from the same group continued the work on MCH using 3 wt % Pt/anatase TiO_2_. The catalyst converted 37% of MCH in an electric field at low temperatures, i.e., 448 K. To apply an electric field, two stainless-steel electrodes contacting the catalyst bed from both sides were used. It seems that proton hopping in the electric field promotes selective MCH dehydrogenation. From the results of XPS and X-ray absorption near edge structure (XANES) analyses, it can be estimated that the Pt’s reducing ability is associated with the formation of metallic Pt. Moreover, the reductive catalyst is essential for the weaker interaction between Pt and the π electrons from the toluene, which does not lead to methane or a carbonaceous byproduct being formed.

Liao et al.^28^ performed the formaldehyde oxidation reaction (FOR) using a custom single-crystal electrode that was made by an annealing-and-quenching method. Voltammetry was used for the evaluation of the electrochemical activity on Cu(111), Ni(111), and Cu_98.5_Ni_1.5_(111) electrodes in KOH. They concluded that Ni(111) is not suitable as a catalyst due to the dehydrogenation of HCHO into CHO species, which adsorbed on the electrode and caused passivation. Nevertheless, the presence of a tiny amount of Ni as a dopant boosts the peak current of a Cu electrode in the FOR reaction by 5-fold. From the Tafel plot, the researchers determined the FOR exchange current densities for Cu_98.5_Ni_1.5_(111) and Cu(111), yielding 547 and 49 μA cm^2^, respectively. This indicated a 12-fold rise in the FOR rate at the equilibrium potential. A comparison with previous results illustrates the benefit of using single-crystal electrodes in interfacial structure studies due to the possibility of dealloying during the processes, leading to different structures at the interface and the bulk. XPS analysis reveals that Ni atoms submerge into the bulk of the electrode, thereby forming a 3–5-atomic-layer-thick, Cu-rich interface.^29^ Increasing the Ni content did not lead to a higher FOR activity. DFT calculations support the results, revealing that the Ni dopant positioned in the second layer of the Cu substrate tunes the electronic state of a Cu substrate, facilitating the adsorption of intermediates and the following oxidation reactions.

Yao et al.^30^ successfully converted CO directly to formaldehyde (HCHO) under ambient conditions using the hybrid thermal and electrochemical approach in which MoP was used as the catalyst. According to *in situ* DRIFTS and DFT simulations, the authors propose that the reaction is between dissolved CO and *H species. The latter is generated *in situ* by H_2_ underpotential deposition on MoP active sites. The tuning of the current density and the reaction temperature is required to avoid probable hydrogen-evolution reactions. Previously, they used the half-cell, which comprises a three-electrode system. MoP deposited on a carbon cloth served as the working electrode, and Ag/AgCl and Pt wire were used as the reference and counter electrodes, respectively. A HCHO production rate obtained using the electrochemical and thermal methods was an order of magnitude higher, −1.032 mmol (g_cat_ h)^−1^ compared to the purely thermal catalysis of 0.07–0.1 mmol (g_cat_ h)^−1^. Moreover, they demonstrate CO reduction in an H-type 2-compartment cell containing two electrode configurations separated by an anion-exchange membrane and KOH as the electrolyte. The latter caused a lower HCHO formation rate due to its internal resistance and the nonoptimized process. Nevertheless, they were able to demonstrate spontaneous HCHO production. At zero voltage, the production rate was 6.1 mg (g_cat_ h)^−1^.

**Figure 7:**
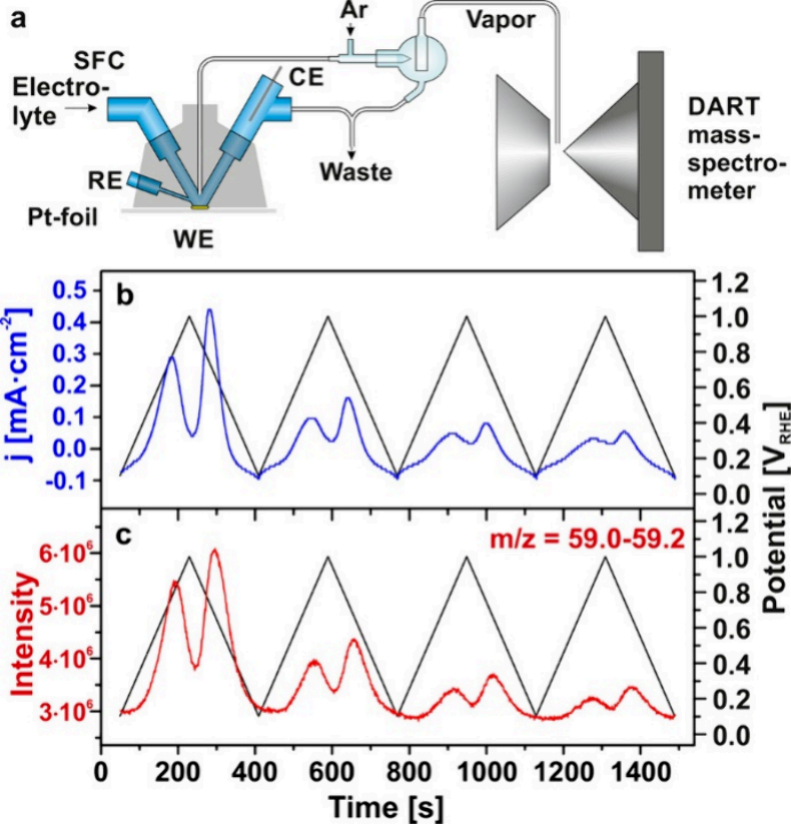
Currents and products recorded during IPA oxidation on polycrystalline Pt monitored by CV in combination with EC-RTMS using SFC coupled with direct analysis in real time (DART). (a) Schematic of the experimental setup. (b) Current density and (c) corresponding DART signal for mass *m*/*z* = 59.1 ± 0.1, 0.2 M IPA in 0.1 M HClO_4_ with a flow rate of 0.5 mL min^–1^ and a scan rate of 10 mV s^–1^. Reprinted with permission from ref (31). Copyright 2020 American Chemical Society.

Waidhas et al.^31^ investigated promising electrofuels for possible future liquid energy-storage technologies (Figure 7). Isopropyl alcohol (IPA) was converted to acetone (ACE) by electrooxidation using three different Pt electrodes, namely, (111) faceted single-crystalline Pt, polycrystalline Pt, and nanostructured tubular Pt electrodes. The techniques of cyclic voltammetry (CV), electrochemical real-time mass spectrometry (EC-RTMS), and electrochemical infrared reflection–absorption spectroscopy (EC-IRRAS) combined with DFT calculations were utilized to study the electrocatalytic activity. Selective oxidation of the IPA to ACE was noted with an onset at 0.3 V_RHE_ when Pt(111) was used, while the formation of adsorbed CO was not observed under any conditions. However, a small amount of CO_2_ was formed in the narrow potential range between 0.8 and 1.0 V_RHE_. Below 0.9 V_RHE_, however, the OH species blocked the active sites required for IPA oxidation. Under certain conditions (between 0.8 and 1.0 V_RHE_), the OH and ACE/IPA species coexist on the electrode’s surface, leading to a complete oxidation to CO_2_. In addition, it was found that cycling to 1.5 V_RHE_ could reactivate the catalyst as the adsorbed species were removed. However, the limited performance of all the electrocatalysts is linked to severe self-poisoning by the adsorbed IPA and ACE. It should be noted that there is room for improvement, with the focus on developing new materials that prevent the blocking of the active sites on the electrodes.

### Electrified Processes Powered by Electricity

#### Microwave-Assisted Catalysis

Microwaves are a form of electromagnetic radiation with wavelengths between 1 m and 1 mm and frequencies between 300 MHz and 300 GHz. When we expose certain materials to microwave frequencies, electromagnetic relaxation occurs. This is a delay in the dielectric constant of the material, caused by the delay in the polarization as it responds to the changing electric or magnetic fields. This relaxation causes friction between the molecules and energy losses that generate heat.^32^

**Figure 8:**
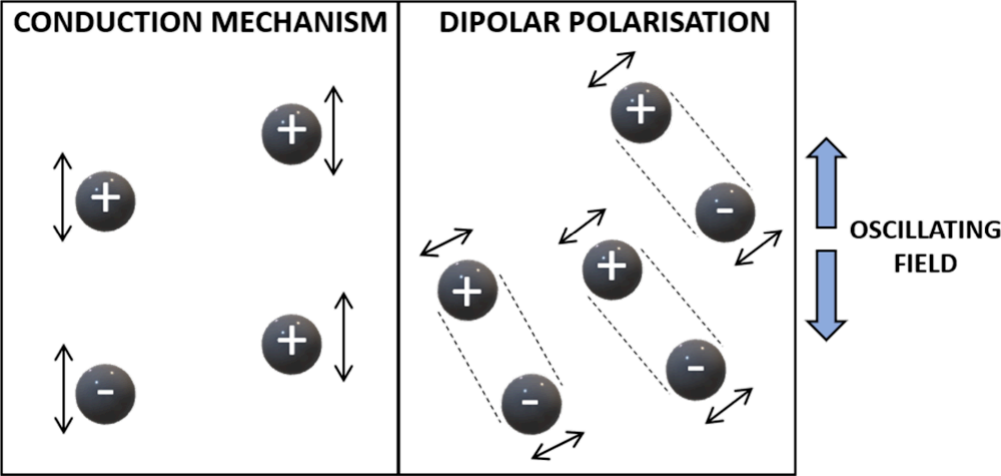
Two different mechanisms of heating: conduction mechanism (left) and dipolar polarization (right).

Microwave-assisted heating/dielectric heating is controlled by two different mechanisms, polarization and ionic conduction in the material (Figure 8), and there are two different mechanisms of heating, conduction mechanism (left) and dipolar polarization (right) (Figure 8).

(a) Ionic conduction: Ions oscillate in response to a changing electric field that generates an electric current. The collision of the charged particles with other molecules causes an internal resistance that leads to current generation and consequently heating.
(b) Polarization: The electric field shifts the charge particles in the material from their equilibrium position and induces dipoles in a high-frequency electromagnetic field. The generated dipoles react to the applied electric field with rotation, which leads to friction and heating.^33^

Recently, interest in MW-supported heterogeneous catalysis has increased due to the enhancement of the thermal effects of MW heating by the generation of hotspots, superheating, and selective heating modes. The aim is to prepare a catalyst that is able to absorb microwaves and heat. It has been found that a nonuniformly distributed electromagnetic field on the catalyst’s surface creates hotspots that facilitate an increase in the reaction rate. Haneishi et al.^34^ showed that the intensity of the electric field is significantly higher in the contact region between two catalyst particles. The temperature gradient between the catalyst and metal site, created in some reactions due to microwave-metal discharge, could benefit the reaction.^35^ The catalyst’s particle morphology affects the material’s dielectric properties and therefore the electric field distribution. An additional advantage of MW-heating is increased selectivity, as the catalyst temperature is much higher than the bulk temperature. This enables a more targeted catalytic reaction. For instance, it is possible to heat a particular part of the catalyst particle to higher temperatures. Thus, MW active material will heat to higher temperatures, causing more selective overheating of the metallic sites and increased reaction rates.^33^

**Figure 9:**
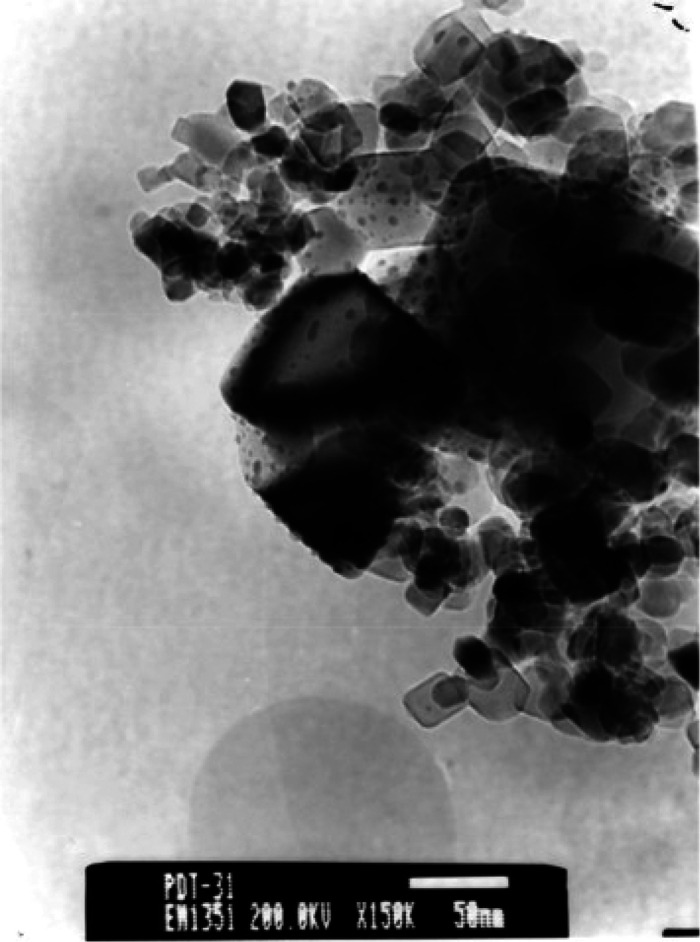
TEM image of the 1% Pd/TiO_2_ sample. Reprinted with permission from ref (20). Copyright 2017 Elsevier.

Kustov et al.^20^ showed a comparison of the dehydrogenation system of perhydro-*N*-ethylcarbazole (H-NEC) in thermal or microwave activation mode using mono- and bimetallic catalysts on a TiO_2_ support. They chose TiO_2_ as a support because it is a good semiconductor that can absorb MW energy. A TEM image of the synthesized monometallic catalyst, Pd-TiO_2_, is shown in Figure 9. The size of the Pd particles varies between 1 and 6 nm. Pd was chosen as the first metal for testing its catalytic activity because it is relatively cheap and can be easily alloyed with metals such as Ru, Pt, Cr, Ni, Ge, and W. The catalytic activity was improved by adding a second metal. In thermal catalysis, Ru and Pt were found to be the leading promoters. Therefore, they were selected for further testing in MW-assisted catalysis (5 W, 5.77 GHz). The latter leads to a 33% decrease in the temperature required for complete conversion, which is also achieved in a shorter time (100 min). While in thermal catalysis for Ru- and Pt-modified catalysts, only 75% conversion was achieved in 374 and 346 min, respectively. Moreover, the formation of CH_4_ and C_2_H_6_ was reduced by selective heating of the metal nanoparticles (NPs) in a relatively cold oxide matrix, which prevented the cracking of organic substrates on the oxide species. The important intermediates of the reaction were identical in both cases. The selectivity for the fully dehydrogenated product, *N*-ethyl-carbazole (NEC), was about 75%. In conclusion, they confirmed that the highly dispersed metal on the support could absorb the energy of the MWs and improve the performance of the catalysts.

#### Magnetic-Assisted Catalysis

Magnetic-assisted catalysis is also an interesting alternative to conventional heating. It is a relatively new development in heterogeneous catalysis, but it has rapidly expanded in recent years. The principle is that, when ferro- and ferrimagnetic NPs are exposed to an external alternating magnetic field, the energy of the alternating magnetic field is locally converted by NPs into heat due to the hysteresis losses. Such advanced technology offers fast, on-demand start-up/shut-down, operation under steady-state conditions limited only by kinetics, avoiding the heat-transfer limitations, etc.^36^

The first report of this application was from the Kirschning group. Ceylan et al.^37^ demonstrated for the first time that magnetic NPs could heat in an alternating current (AC) field and have great potential for laboratory and industrial processes. They drove various important organic reactions (Suzuki–Miyaura and Heck coupling) using silica-coated Fe_3_O_4_/Fe_2_O_3_ NPs of 10–40 nm diameter and decorated with catalytically active palladium.

To the best of our knowledge, this concept has not yet been applied to a LOHC system. However, many articles were published recently demonstrating magnetic-assisted catalysis for several technologically important reactions, i.e., the Fischer–Tropsch reaction, dry and steam reforming of methane/propane, and the Sabatier reaction.^36^ In addition, it has been used in research for the sustainable production of specialty chemicals and biofuels from biomass, such as the hydrogenation of levulinic acid or furfural.^38^ As a heating agent, the most frequently used were iron-based materials (zerovalent iron, iron carbides, and magnetic iron oxides). Those are characterized by the relative ease of the preparation and the abundance of Fe. However, for more demanding applications where a high Curie temperature or oxidation resistance is a prerequisite, alloy NPs consisting of Fe, Co, and/or Ni are available.^36^ Considering successful applications of magnetic heating to drive endothermic and exothermic reactions (hydrogenations as well), as described in previously published research, we can, without reservation, claim that magnetic heating of catalysts will be applied to LOHC (de)hydrogenation reactions in the near future.

#### Plasma-Assisted Catalysis

Plasma catalysis is also an emerging field at the interface between plasma research and catalysis. Plasmas represent an energy source consisting of reactive ionized species such as ions, radicals, and excited molecules that can be used to activate and transform molecules on catalyst surfaces and to promote or modify chemical reactions. Plasma catalysis offers advantages such as lower temperatures, shorter reaction times, and better selectivity and efficiency compared to conventional catalytic processes.^39^ Yan et al.^40^ provided a concise overview of recent advances in plasma catalysis, particularly with respect to LOHC. Their review covers several technologically important processes, including NH_3_ synthesis, Fischer–Tropsch reactions, methane reforming, etc. For this reason, we believe that using plasma in an electrified LOHC system has potential and points to a promising future for this technology.

Table 2 shows a comparison of the different approaches to catalyze the reaction. Each of them has some advantages and disadvantages. Unlike the other two methods, thermos-catalysis has a high conversion rate. Since the processes are technologically developed and established, they are also easier to apply on a larger scale. Unfortunately, they are challenging to electrify using renewable electricity. Typically, the reactors are heated by an external heat source, such as gas-fired or resistive heaters surrounded by insulation. The large heat capacity of such systems prevents the rapid start-ups and ramp-downs inevitably associated with the utilization of intermittent renewable electricity. This could be overcome, to some extent, by coupling a large-capacity battery with a resistively heated reactor. However, additional costs may make the system economically less favorable. In addition, the rapid response of a reactor will still not be achieved. A rapid response could be beneficial for using the technology in future filling stations where on-demand hydrogen release is needed to fill fuel-powered electric vehicles. The key overarching challenge of electrification is the availability, stability, and costs of renewable energy sources, compared to fossil fuels. There are also technical scalability barriers, which will be overcome, as CO_2_ emissions will become ever more expensive.

**Table 2:** Summary and Comparison of Emerging Processes^a^

Ref.	Catalysis	Reaction	*T* [°C]	Conversion [%]	Time [min]	Conditions	Advantages +	Disadvantages –
Thermal Catalysis								
(41)	Pt-TiO_2_	H18-DBT dehydrogenation	310	90	130	1 bar		
(42)	Pt-zeolite beta	MCH dehydrogenation	360	83	N/A	1 bar	high turnover rate	high *T*
(42)	Pt-zeolite beta	Toluene hydrogenation	285	91	N/A	1 bar	easier scale-up	not compatible with green energy (intermittent)
(20)	Ru–Pd/TiO_2_	H-NEC dehydrogenation	195	75	374	1 bar	established processes	
(20)	Pt–Pd/TiO_2_	H-NEC dehydrogenation	195	75	346	1 bar		
MW-Assisted Catalysis								
(20)	Ru–Pd/TiO_2_	H-NEC dehydrogenation	180–190	100	110	5 W, 5.77 GHz	better selectivity	MW absorbing catalysts needed
(20)	Pt–Pd/TiO_2_	H-NEC dehydrogenation	180–190	100	100	5 W, 5.77 GHz	shorter time rapid start up and shut down	
Electrochemical Catalysis								
(30)	MoP/CC	CO hydrogenation	20	1.032 mmol (g_CAT_ h)^−1^	N/A	50% FE	easily compatible with green energy	lower productivity
(27)	Pt/TiO_2_	MCH dehydrogenation	175	37	N/A	5.0 mA	lower pressures and temperatures	
(22)	Pt/CeO_2_	MCH dehydrogenation	150	21.6^b^	N/A	3.0 mA		

a H18-DBT = perhydro-dibenzyltoluene; MCH = methylcyclohexane; H-NEC = perhydro-*N*-ethylcarbazole; FE = Faradaic efficiency; CC = carbon cloth; N/A = not applicable.

b H_2_ yield.

Electrochemical processes are purely electrical and operate at lower temperatures and pressures. Microwave-assisted catalysis involves using catalysts made of metals that can absorb microwaves. This method improves the selectivity as specific parts of the catalyst NPs (metal) are heated more than the others (support). This allows for complete conversion to occur in a shorter time compared with conventional heating methods. Additionally, this method provides for on-demand ignition and operation. However, further research and development are needed in catalyst materials and technologies. For example, to utilize MW and magnetically heated catalysis, new catalysts absorbing microwaves or converting alternating magnetic fields to heat need to be developed. Typical catalysts such as Pt, Ru, and Pd are not ferromagnetic; i.e., they cannot convert the alternating magnetic field to heat and need to be coupled with ferromagnetic NPs. Those need to be optimized to efficiently heat specific alternating magnetic fields and frequency efficiently. Therefore, such catalysts are nanocomposite materials in which ferromagnetic and catalytic NPs must be optimized for their particular role. In addition, reactors in both cases have to be constructed out of MW and alternating magnetic field nonabsorbing material to allow specific heating of the catalyst. At the same time, they must withstand conditions such as temperatures in the range of 100 to 300 °C, reducing conditions, and elevated pressure, to mention just a few. To meet the requirements, the traditional materials of choice for constructing chemical reactors, such as stainless steel, must be omitted. As for every catalytic transformation, not only is the activity of the catalysts important but also their stability under realistic operational conditions. To the best of our knowledge, no stability tests were performed or reported. Because the field is emerging, it is expected that researchers will focus more on proof-of-concept research than on research shedding light on engineering aspects. We encourage the community to address this critical issue in future research. Advances will come from researching the scalability, robustness, and economics of electrification technologies. However, for many of those indicated, even when renewables are accessible, a benchmarking with traditional thermal catalysis will still be unfavorable.

## Conclusion

In conclusion, utilizing liquid organic hydrogen carriers (LOHCs) as vectors for hydrogen transport and energy storage is a relatively new but immensely promising technology. It can potentially mitigate the intermittent nature of renewable energy sources through electrifying and decarbonizing the LOHC (de)hydrogenation processes. This leads to the possibility of easier downscaling of H_2_ storage/release processes while still being economical and thus enabling the decentralized utilization of intermittent renewable electricity. The benefit of electrochemical processes is that they can operate at lower temperatures, even at room temperature in some cases. However, they still suffer from low productivity. Another issue is the poisoning (reversible in some cases) of the electrodes, which reduces productivity. The benefits of microwave (MW)-heated processes are the relatively rapid heating time and the rapid cooling after the MWs are switched off. This enables, at least in principle, smaller modular systems for energy storage. Additionally, the short start-up and shut-down times combine well with the intermittent nature of renewables. Similar benefits are expected from the magnetic (induction) heating of novel catalysts (which contain magnetic nanoparticles that heat upon exposure to alternating magnetic fields). Both approaches add contactless and rapid heating, therefore making the thermocatalytic process responsive and adaptable to intermittent renewable electricity supply. All the mentioned processes are still in their infancy with respect to their application in LOHC (de)hydrogenation, mainly due to the lack of suitable catalysts. We anticipate that the field will advance through the rational design of novel catalysts and the evaluation of processes with the strong involvement of ever-advancing fields of multiscale modeling and machine learning.
